# Eigenvalues of the resistance-distance matrix of complete multipartite graphs

**DOI:** 10.1186/s13660-017-1570-1

**Published:** 2017-11-28

**Authors:** Kinkar Chandra Das, Yujun Yang

**Affiliations:** 1 Department of Mathematics, Sungkyunkwan University, Suwon, 440-746 Republic of Korea; 2 School of Mathematics and Information Science, Yantai University, Yantai, 264005 P.R. China

**Keywords:** resistance distance, resistance-distance matrix, largest resistance-distance eigenvalue, second largest resistance-distance eigenvalue

## Abstract

Let $G=(V, E)$ be a simple graph. The resistance distance between $i,j\in V$, denoted by $r_{ij}$, is defined as the net effective resistance between nodes *i* and *j* in the corresponding electrical network constructed from *G* by replacing each edge of *G* with a resistor of 1 Ohm. The resistance-distance matrix of *G*, denoted by $R(G)$, is a $\vert V \vert \times \vert V \vert $ matrix whose diagonal entries are 0 and for $i\neq j$, whose *ij*-entry is $r_{ij}$. In this paper, we determine the eigenvalues of the resistance-distance matrix of complete multipartite graphs. Also, we give some lower and upper bounds on the largest eigenvalue of the resistance-distance matrix of complete multipartite graphs. Moreover, we obtain a lower bound on the second largest eigenvalue of the resistance-distance matrix of complete multipartite graphs.

## Introduction

Throughout the paper we consider only simple graphs, that is, graphs without loops and multi-edges. Let $G=(V, E)$ be a connected graph with a vertex set $V=\{1,2,\ldots,n\}$ and an edge set $E=E(G)$. The resistance distance [1] between any two vertices *i* and *j*, denoted by $r_{ij}$, is defined as the net effective resistance between nodes *i* and *j* in the corresponding electrical network constructed from *G* by replacing each edge with a resistor of 1 Ohm. The resistance-distance matrix of *G*, denoted by $R(G)$, is a $\vert V \vert \times \vert V \vert $ matrix whose diagonal entries are 0 and for $i\neq j$, whose *ij*-entry is $r_{ij}$. Let $\rho_{1}\geq\rho_{2}\geq\cdots\geq\rho_{n}$ denote the eigenvalues of $R(G)$. They are usually called the resistance-distance eigenvalues of *G*. In recent years, much study has been done on resistance distances. For more information, the readers are referred to most recent papers [2–12] and the references therein. In this paper, we study the resistance-distance matrix of complete multipartite graphs. The paper is organized as follows. In Section 2, we compute resistance distances in complete multipartite graphs. In Section 3, we determine the eigenvalues of the resistance distance matrix of complete multipartite graphs. In Section 4, we give some lower and upper bounds on the largest eigenvalue of the resistance-distance matrix of complete multipartite graphs. In Section 5, we obtain a lower bound on the second largest eigenvalue of the resistance-distance matrix of complete multipartite graphs.

## Resistance distances in complete multipartite graphs

In this section, we compute resistance distances between any pair of vertices in the complete multipartite graph $K_{n_{1}, n_{2},\ldots, n_{k}}$ via electrical network approach. Recall that *G* is a complete *k*-partite graph if the vertex set *V* can be partitioned into *k* parts $V_{1}, V_{2}, \ldots,V_{k}$ such that $uv\in E(G)$ if and only if *u* and *v* are in different parts. If $\vert V_{i} \vert =n_{i}$ ($i=1,2,\ldots,k$), then *G* is denoted by $K_{n_{1}, n_{2},\ldots, n_{k}}$. The following two lemmas play essential roles further.

### Lemma 2.1

([13])

*Let* $i, j$ *be vertices of* *G* *satisfying that they have the same neighborset* *N* *in* $V/\{i,j\}$. *Then*

1 $$ r_{ij}= \textstyle\begin{cases} \frac{2}{ \vert N \vert +2} & \textit{if }ij\in E(G),\\ \frac{2}{ \vert N \vert } & \textit{otherwise}. \end{cases} $$

### Lemma 2.2

([13] (The reduction principle))

*If* $S\subset V$ *satisfies that all vertices in* *S* *have the same neighborset* *N* *in* $G-S$. *Let* *H* *be the graph obtained from* $G[S\cup N]$ *by deleting all the edges between vertices in* *N*. *Then the resistance distance between any two vertices of* *S* *in* *G* *is the same as the resistance distance between them in* *H*.

Now we are ready to give the main result of this section.

### Theorem 2.3

*Resistance distances in* $K_{n_{1}, n_{2},\ldots, n_{k}}$ *can be computed as follows*:

2 $$ r_{uv}= \textstyle\begin{cases} \frac{2}{n-n_{i}} & \textit{if }u, v\in V_{i},\\ \frac{(n-1)(2n-n_{i}-n_{j})}{n [n^{2}-(n_{i}+n_{j})n+n_{i} n_{j} ]} & \textit{if }u\in V_{i}, v\in V_{j}, \textit{ and } i\neq j. \end{cases} $$

### Proof

For $u,v\in V_{i}$, it is easily seen that *u* and *v* have the same neighborset *N* with $\vert N \vert =n-n_{i}$. Hence, by Lemma 2.1, we have

$$r_{uv}=\frac{2}{n-n_{i}}, $$

as required.

**Figure 1:**
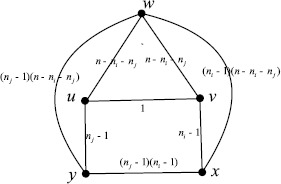
**The simplified graph** $\pmb{G^{*}}$ **.**

Now suppose that $u\in V_{i}$ and $v\in V_{j}$. Let $S=V_{i}\cup V_{j}$ and $N=V-S$. Let $G^{*}$ be the graph obtained from $K_{n_{1}, n_{2},\ldots, n_{k}}$ by deleting all the edges between vertices in *N*. Then, by the reduction principle, the resistance distance between *u* and *v* in $K_{n_{1}, n_{2},\ldots, n_{k}}$ is equal to the resistance distance between *u* and *v* in $G^{*}$. In what follows, we compute the resistance distance between *u* and *v* in $G^{*}$. If we apply a unit potential across *u* and *v*, then by symmetry, all the vertices in $V_{i}\setminus\{u\}$ have the same potential, all the vertices in $V_{j}\setminus\{v\}$ have the same potential, and all the vertices in *N* have the same potential. In an electrical point of view, vertices that have the same potential can be regarded as identical so that they can be shortened together. Consequently, we shorten all the vertices in $V_{i}\setminus\{ u\}$ together to get a new vertex *x*, shorten all the vertices in $V_{j}\setminus\{v\}$ together to get a new vertex *y*, and shorten all the vertices in *N* together to get a new vertex *w*. Then $G^{*}$ can be simplified to the network $\mathcal{N}$ as shown in Figure 1, where the weight $c_{ab}$ on each edge *ab* denotes the edge conductance (i.e., reciprocal of edge resistance).

Now apply a unit potential across *u* and *v* in $\mathcal{N}$ and suppose the absolute potentials of *u*, *v*, *x*, *y*, *w* are $V_{u}=1$, $V_{v}=0$, $V_{x}$, $V_{y}$, $V_{w}$, respectively. Then, by Kirchhoff’s laws, we have

$$\begin{aligned} &(V_{x}-V_{y})c_{xy}+(V_{x}-V_{v})c_{xv}+(V_{x}-V_{w})c_{xw}=0, \\ &(V_{y}-V_{x})c_{xy}+(V_{y}-V_{u})c_{yu}+(V_{y}-V_{w})c_{yw}=0, \\ &(V_{w}-V_{u})c_{wu}+(V_{w}-V_{x})c_{xw}+(V_{w}-V_{y})c_{yw}+(V_{w}-V_{v})c_{wv}=0. \end{aligned}$$

Then simple calculation shows that

3 $$\begin{aligned} &V_{x}=\frac {(n_{i}-1)(n_{j}-1)V_{y}+(n_{i}-1)(n-n_{i}-n_{j})V_{w}}{n_{i}-1+(n_{i}-1)(n_{j}-1)+(n_{i}-1)(n-n_{i}-n_{j})}, \end{aligned}$$

4 $$\begin{aligned} &V_{y}=\frac {n_{j}-1+(n_{i}-1)(n_{j}-1)V_{x}+(n_{j}-1)(n-n_{i}-n_{j})V_{w}}{n_{j}-1+(n_{i}-1)(n_{j}-1)+(n_{j}-1)(n-n_{i}-n_{j})}, \end{aligned}$$

5 $$\begin{aligned} &V_{w}=\frac {n-n_{i}-n_{j}+(n_{i}-1)(n-n_{i}-n_{j})V_{x}+(n_{j}-1)(n-n_{i}-n_{j})V_{y}}{2(n-n_{i}-n_{j})+(n_{i}-1)(n-n_{i}-n_{j})+(n_{j}-1)(n-n_{i}-n_{j})}. \end{aligned}$$

Solving the above linear system, we get

$$\begin{aligned} &V_{x}=\frac{n(n_{j}-1)+(n-1)(n-n_{i}-n_{j})}{(2n-n_{i}-n_{j})(n-1)}, \\ &V_{y}=\frac{n(n-n_{i})}{(2n-n_{i}-n_{j})(n-1)}, \\ &V_{w}=\frac{n-n_{i}}{2n-n_{i}-n_{j}}. \end{aligned}$$

Denote the total current flows from *u* to *v* by *I*. Then, by Ohm’s law, we have

$$\begin{aligned} r_{uv}&=\frac{V_{u}-V_{v}}{I}=\frac{1-0}{I} \\ &=\frac{1}{(V_{u}-V_{w})(n-n_{i}-n_{j})+(V_{u}-V_{y})(n_{j}-1)+(V_{u}-V_{v})\times 1} \\ &=\frac{1}{ { (1-\frac{n-n_{i}}{2n-n_{i}-n_{j}} )(n-n_{i}-n_{j})+ [1-\frac{n(n-n_{i})}{(2n-n_{i}-n_{j})(n-1)} ](n_{j}-1)+1}} \\ &=\frac{(n-1)(2n-n_{i}-n_{j})}{n [n^{2}-(n_{i}+n_{j})n+n_{i} n_{j} ]}, \end{aligned}$$

as required. □

### Remark

It should be mentioned that resistance distances in complete multipartite graphs have also been determined in [10] via an alternative method.

By Theorem 2.3, for simplicity, in the following, we use $r_{i}$ to denote the resistance distance between any two vertices in $V_{i}$, and use $r_{ij}$ to denote the resistance distance between any $u\in V_{i}$ and $v\in V_{j}$. Thus the resistance-distance matrix $R(K_{n_{1}, n_{2},\ldots, n_{k}})$ of $K_{n_{1}, n_{2},\ldots, n_{k}}$ is

$$ \begin{pmatrix} r_{1} J_{n_{1}, n_{1}}-r_{1} I_{n_{1}} & r_{12} J_{n_{1}, n_{2}} & r_{13} J_{n_{1}, n_{3}} & \ldots& r_{1k} J_{n_{1}, n_{k}} \\ r_{12} J_{n_{2}, n_{1}} & r_{2} J_{n_{2}, n_{2}}-r_{2} I_{n_{2}} & r_{23} J_{n_{2}, n_{3}} & \ldots& r_{2k} J_{n_{2}, n_{k}} \\ \vdots& \vdots& \vdots& \ldots& \vdots\\ r_{1k} J_{n_{k}, n_{1}} & r_{2k} J_{n_{k}, n_{2}} & r_{3k} J_{n_{k}, n_{3}} & \ldots& r_{k} J_{n_{k}, n_{k}}-r_{k} I_{n_{k}} \end{pmatrix}, $$

where $J_{st}$ denotes the $s\times t$ matrix of all ones, $I_{l}$ denotes the identity matrix of order *l*. In what follows, we always write $R(K_{n_{1}, n_{2},\ldots, n_{k}})$ to *R* for short.

## The eigenvalues of the resistance-distance matrix of complete multipartite graphs

In this section we obtain the eigenvalues of the resistance-distance matrix of complete *k*-partite graphs $K_{n_{1}, n_{2},\ldots, n_{k}}$.

### Theorem 3.1

*Let* *G* *be a complete* *k*-*partite graph* $K_{n_{1}, n_{2},\ldots, n_{k}}$ *on* *n* *vertices*. *Then the characteristic polynomial of* *R* *is*

$$R_{G}(x)=\det(xI_{n}-R)=\prod^{k}_{i=1}(x+r_{i})^{n_{i}-1} \vert xI_{k}-D_{2} \vert , $$

*where*

6 $$ D_{2}= \begin{pmatrix} (n_{1}-1)r_{1} & n_{2} r_{12} & n_{3} r_{13}& \cdots& n_{k} r_{1k} \\ n_{1} r_{12} & (n_{2}-1)r_{2} & n_{3} r_{23}&\cdots& n_{k} r_{2k} \\ \vdots& \vdots&\vdots&\ddots& \vdots\\ n_{1} r_{1k} & n_{2} r_{2k} & n_{3} r_{3k} &\cdots& (n_{k}-1)r_{k} \end{pmatrix}. $$

### Proof

As given above, the resistance-distance matrix of $K_{n_{1}, n_{2},\ldots, n_{k}}$ is

$$R= \begin{pmatrix} r_{1} J_{n_{1}, n_{1}}-r_{1} I_{n_{1}} & r_{12} J_{n_{1}, n_{2}} & r_{13} J_{n_{1}, n_{3}} & \ldots& r_{1k} J_{n_{1}, n_{k}} \\ r_{12} J_{n_{2}, n_{1}} & r_{2} J_{n_{2}, n_{2}}-r_{2} I_{n_{2}} & r_{23} J_{n_{2}, n_{3}} & \ldots& r_{2k} J_{n_{2}, n_{k}} \\ \vdots& \vdots& \vdots& \ldots& \vdots\\ r_{1k} J_{n_{k}, n_{1}} & r_{2k} J_{n_{k}, n_{2}} & r_{3k} J_{n_{k}, n_{3}} & \ldots& r_{k} J_{n_{k}, n_{k}}-r_{k} I_{n_{k}} \end{pmatrix}. $$

Hence, by linear algebra knowledge,

$$\begin{aligned} R_{G}(x)={}&\det(x I_{n}-R) \\ ={}&\left \vert \begin{matrix} (x+r_{1})I_{n_{1}}-r_{1} J_{n_{1}, n_{1}} & -r_{12} J_{n_{1}, n_{2}} & -r_{13} J_{n_{1}, n_{3}} & \ldots& -r_{1k} J_{n_{1}, n_{k}} \\ -r_{12} J_{n_{2}, n_{1}} & (x+r_{2})I_{n_{2}}-r_{2} J_{n_{2}, n_{2}} & -r_{23} J_{n_{2}, n_{3}} & \ldots& -r_{2k} J_{n_{2}, n_{k}} \\ \vdots& \vdots& \vdots& \ldots& \vdots\\ -r_{1k} J_{n_{k}, n_{1}} & -r_{2k} J_{n_{k}, n_{2}} & -r_{3k} J_{n_{k}, n_{3}} & \ldots& (x+r_{k})I_{n_{k}}-r_{k} J_{n_{k}, n_{k}} \end{matrix} \right \vert \\ ={}&\prod^{k}_{i=1}(x+r_{i})^{n_{i}-1} \left \vert \begin{matrix} x-(n_{1}-1)r_{1} & -n_{2} r_{12} & -n_{3} r_{13}& \cdots& -n_{k} r_{1k} \\ -n_{1} r_{12} & x-(n_{2}-1)r_{2} & -n_{3} r_{23}&\cdots& -n_{k} r_{2k} \\ \vdots& \vdots&\vdots&\ddots& \vdots\\ -n_{1} r_{1k} & -n_{2} r_{2k} & -n_{3} r_{3k} &\cdots& x-(n_{k}-1)r_{k} \end{matrix} \right \vert . \end{aligned}$$

This completes the proof. □

### Corollary 3.2

*Let* $G=K_{n_{1}, n_{2},\ldots, n_{k}}$. *Then the eigenvalues for the resistance*-*distance matrix of* *G* *are* $-r_{i}$ *of multiplicity* $n_{i}-1$ $(i=1, 2,\ldots, k)$ *and the remaining eigenvalues satisfy the following*:

$$\vert xI_{k}-D_{2} \vert =0, $$

*where* $D_{2}$ *is given by* (6).

### Corollary 3.3

*The largest eigenvalue of the resistance*-*distance matrix of complete* *k*-*partite graph* $K_{n_{1}, n_{2},\ldots, n_{k}}$ *is given by*

$$\vert xI_{k}-D_{2} \vert =0. $$

## Lower and upper bounds on the largest eigenvalue of the resistance-distance matrix of complete multipartite graphs

In this section we give some lower and upper bounds on $\rho_{1}(G)$ of complete *k*-partite graph $K_{n_{1}, n_{2},\ldots, n_{k}}$. For this we need the following two results.

### Lemma 4.1

([14])

*If* $q_{1},q_{2},\ldots,q_{n}$ *are positive numbers*, *then*

$$ \min_{i} \frac{p_{i}}{q_{i}}\leq\frac{p_{1}+p_{2}+\cdots+p_{n}}{q_{1}+q_{2}+\cdots +q_{n}} \leq\max _{i} \frac{p_{i}}{q_{i}} $$

*for any real numbers* $p_{1},p_{2},\ldots,p_{n}$. *Equality holds on both sides if and only if all the ratios* ${\frac{p_{i}}{q_{i}}}$ *are equal*.

Now we obtain the spectral radius for the resistance-distance matrix of $K_{n_{1}, n_{1},\ldots, n_{1}}$.

### Lemma 4.2

*Let* *G* *be a complete* *k*-*partite graph* $K_{n_{1}, n_{1},\ldots, n_{1}}$ *of order* *n*. *Then*

$$\rho_{1}(G)=\frac{2}{n-n_{1}} \biggl[n_{1}-1+ \frac{n_{1}(k-1)(n-1)}{n} \biggr]. $$

*Furthermore*, *all the remaining eigenvalues are* $- \frac{2}{n-n_{1}}$ *and* $- \frac{2}{n}$, *with multiplicities* $n-k$ *and* $k-1$, *respectively*.

### Proof

For $G=K_{n_{1}, n_{1},\ldots, n_{1}}$, by Theorem 2.3, we have $r_{i}=\frac{2}{n-n_{1}}\ (i=1, 2,\ldots, k) \mbox{ and } r_{ij}=\frac {n-1}{n} (\frac{1}{n-n_{1}}+\frac{1}{n-n_{1}} ) \mbox{ for all } i \mbox{ and } j, i\neq j$. Since

$$D_{2}= \begin{pmatrix} (n_{1}-1)r_{1} & n_{1} r_{12} & n_{1} r_{12}& \cdots& n_{1} r_{12} \\ n_{1} r_{12} & (n_{1}-1)r_{1} & n_{1} r_{12}&\cdots& n_{1} r_{12} \\ \vdots& \vdots&\vdots&\ddots& \vdots\\ n_{1} r_{12} & n_{1} r_{12} & n_{1} r_{12} &\cdots& (n_{1}-1)r_{1} \end{pmatrix}, $$

it is easily obtained that the eigenvalues of $D_{2}$ are

$$(n_{1}-1)r_{1}+(k-1)r_{12}, \underbrace{(n_{1}-1)r_{1}-n_{1}r_{12}, \ldots, (n_{1}-1)r_{1}-n_{1}r_{12}}_{k-1}. $$

Simple calculation shows that

$$(n_{1}-1)r_{1}+(k-1)r_{12}=\frac{2}{n-n_{1}} \biggl[n_{1}-1+\frac {n_{1}(k-1)(n-1)}{n} \biggr] \quad\mbox{and}\quad (n_{1}-1)r_{1}-n_{1}r_{12}=- \frac{2}{n}. $$

Thus it follows that

$$\rho_{1}(G)=\frac{2}{n-n_{1}} \biggl[n_{1}-1+ \frac{n_{1}(k-1)(n-1)}{n} \biggr]. $$

Together with the result in Theorem 2.3, we conclude that eigenvalues other than $\rho_{1}(G)$ are

$$\underbrace{-\frac{2}{n},\ldots,-\frac{2}{n}}_{k-1}, \underbrace{-\frac {2}{n-n_{1}},\ldots,-\frac{2}{n-n_{1}}}_{n-k}. $$

 □

### Theorem 4.3

*Let* *G* *be a complete* *k*-*partite graph* $K_{n_{1}, n_{2},\ldots, n_{k}}$ *of order* *n* *with* $n_{1}\geq n_{2}\geq\cdots \geq n_{k}$. *Then*

7 $$ \frac{2}{n-n_{k}} \biggl[n_{k}-1+\frac{n_{k}(k-1)(n-1)}{n} \biggr]\leq\rho _{1}(G)\leq\frac{2}{n-n_{1}} \biggl[n_{1}-1+ \frac{n_{1}(k-1)(n-1)}{n} \biggr]. $$

*Moreover*, *the equality holds in both sides if and only if* $n_{1}=n_{2}=\cdots=n_{k}$.

### Proof

Let $\mathbf{x}=(x_{1}, x_{2},\ldots, x_{n})^{T}$ be an eigenvector corresponding to the eigenvalue $\rho_{1}(G)$ of $D_{2}$. Then we have

8 $$ D_{2}\mathbf{x}=\rho_{1}(G)\mathbf{x}. $$

From the *i*th equation of (8), we get

9 $$\begin{aligned} \rho_{1}(G)x_{i}&=(n_{i}-1) r_{i} x_{i}+\sum^{k}_{j=1,j\neq i} n_{j} r_{ij} x_{j} \\ &=\frac{2(n_{i}-1)}{n-n_{i}} x_{i}+\frac{n-1}{n} \sum ^{k}_{j=1,j\neq i} \biggl[\frac{1}{n-n_{i}}+ \frac{1}{n-n_{j}} \biggr] n_{j} x_{j} \\ &\geq\frac{2(n_{k}-1)}{n-n_{k}} x_{i}+\frac{2(n-1)}{n (n-n_{k})} \sum ^{k}_{j=1,j\neq i} n_{j} x_{j} \quad\mbox{as }n_{i}, n_{j}\geq n_{k}. \end{aligned}$$

Taking summation on both sides from $i=1$ to *k*, we get

$$\begin{aligned} \rho_{1}(G)\sum^{k}_{i=1}x_{i} \geq&\frac{2(n_{k}-1)}{n-n_{k}} \sum^{k}_{i=1} x_{i}+\frac{2(n-1) (k-1)}{n (n-n_{k})} \sum^{k}_{i=1} n_{i} x_{i}. \end{aligned}$$

By Lemma 4.1, we get

10 $$ \frac{\sum^{k}_{i=1} n_{i} x_{i}}{\sum^{k}_{i=1} x_{i}}\geq n_{k}. $$

From the above two results, we get

$$\rho_{1}(G)\geq\frac{2(n_{k}-1)}{n-n_{k}}+\frac{2(n-1) (k-1)}{n (n-n_{k})} n_{k}= \frac{2}{n-n_{k}} \biggl[n_{k}-1+\frac{n_{k}(k-1)(n-1)}{n} \biggr]. $$

Since $n_{i}, n_{j}\leq n_{1}$ and

$$\frac{\sum^{k}_{i=1} n_{i} x_{i}}{\sum^{k}_{i=1} x_{i}}\leq n_{1}, $$

similarly, from the above, we get

$$\rho_{1}(G)\leq\frac{2}{n-n_{1}} \biggl[n_{1}-1+ \frac{n_{1}(k-1)(n-1)}{n} \biggr]. $$

First part of the proof is done.

Now suppose that the left-hand side equality holds in (7). Then all the inequalities above must be equalities. From the equality in (9), we get $n_{1}=n_{2}=\cdots =n_{k}$. From the equality in (10), we get $n_{1}=n_{2}=\cdots=n_{k}$. Similarly, if the right-hand side equality holds in (7), then we have $n_{1}=n_{2}=\cdots=n_{k}$.

Conversely, let $G\cong K_{n_{1}, n_{1},\ldots, n_{1}}$. By Lemma 4.2, equalities on both sides hold in (7). □

Now we give another upper bound on $\rho_{1}(G)$ of complete *k*-partite graph $K_{n_{1}, n_{2},\ldots, n_{k}}$.

### Theorem 4.4

*Let* *G* *be a complete* *k*-*partite graph* $K_{n_{1}, n_{2},\ldots, n_{k}}$ *of order* *n* *with* $n_{1}\geq n_{2}\geq\cdots\geq n_{k}$. *Then*

11 $$\begin{aligned} \rho_{1}(G)\leq{}&\frac{2(n_{1}-1)}{n-n_{1}}+\frac{n_{1} (n-1)^{2} (k-1)}{n (n-n_{1}) (n_{k}-1)}- \frac{(n-1)(n-n_{1})}{n (n_{1}-1)} \\ &{} +\frac{(n-1)(n-n_{k}) (k-1)}{n (n_{k}-1)} \biggl[1-\frac {(2n-1)}{n-n_{1}}+\frac{n(n-1)}{(n-n_{1})^{2}} \biggr] \end{aligned}$$

*with equality holding if and only if* $n_{1}=n_{2}=\cdots=n_{k}$.

### Proof

Let $\mathbf{x}=(x_{1}, x_{2},\ldots, x_{n})^{T}$ be an eigenvector corresponding to the eigenvalue $\rho_{1}(G)$ of $C^{-1}D_{2}C$, where $C=\mbox{diag}((n_{1}-1) r_{1}, (n_{2}-1) r_{2},\ldots, (n_{k}-1) r_{k})$. Then we have

12 $$ C^{-1}D_{2}C\mathbf{X}=\rho_{1}(G)\mathbf{X} . $$

We can assume that $x_{i}=1$ and $x_{k}\leq1$ for all *k*. From the *i*th equation of (12), we get

$$ \rho_{1}(G)x_{i}=(n_{i}-1) r_{i} x_{i}+\sum^{k}_{j=1,j\neq i} \frac{(n_{j}-1) r_{j} n_{j} r_{ij}}{(n_{i}-1) r_{i}} x_{j}, $$

that is,

13 $$\begin{aligned} \rho_{1}(G)&\leq (n_{i}-1) r_{i}+\sum ^{k}_{j=1,j\neq i}\frac{(n_{j}-1) r_{j} n_{j} r_{ij}}{(n_{i}-1) r_{i}} \end{aligned}$$

14 $$\begin{aligned} &=\frac{2(n_{i}-1)}{n-n_{i}}+\sum^{k}_{j=1,j\neq i} \frac{(n-1) (n_{j}-1) n_{j}}{n (n_{i}-1)} \biggl[\frac{1}{n-n_{j}}+\frac{n-n_{i}}{(n-n_{j})^{2}} \biggr] . \end{aligned}$$

Since

$$ \frac{n_{j}(n_{j}-1)}{n-n_{j}}=1-n-n_{j}+\frac{n(n-1)}{n-n_{j}} $$

and

$$ \frac{n_{j}(n_{j}-1)}{(n-n_{j})^{2}}=1-\frac{2n-1}{n-n_{j}}+\frac {n(n-1)}{(n-n_{j})^{2}}, $$

from (14), we get

$$\begin{aligned} \rho_{1}(G)\leq{}& \frac{2(n_{i}-1)}{n-n_{i}}+\frac{n-1}{n (n_{i}-1)} \sum ^{k}_{j=1,j\neq i} \biggl[1-n-n_{j}+ \frac{n(n-1)}{n-n_{j}} \biggr]+\frac{(n-1) (n-n_{i})}{n (n_{i}-1)} \\ &{}\times\sum^{k}_{j=1,j\neq i} \biggl[1- \frac{2n-1}{n-n_{j}}+\frac {n(n-1)}{(n-n_{j})^{2}} \biggr]. \end{aligned}$$

Since

$$f(x)=\frac{n(n-1)}{(n-x)^{2}}-\frac{2n-1}{(n-x)} $$

is an increasing function on *x*, from the above, we get

15 $$\begin{aligned} \rho_{1}(G)\leq{}& \frac{2(n_{i}-1)}{n-n_{i}}+\frac{n-1}{n (n_{i}-1)} \biggl[-(n-1) (k-1)-(n-n_{i})+\frac{n(n-1)(k-1)}{n-n_{1}} \biggr] \\ &{} +\frac{(n-1) (n-n_{i})}{n (n_{i}-1)} \biggl[1-\frac {2n-1}{n-n_{1}}+\frac{n(n-1)}{(n-n_{1})^{2}} \biggr] (k-1) \\ ={}&\frac{2(n_{i}-1)}{n-n_{i}}+\frac{n_{1} (n-1)^{2} (k-1)}{n (n-n_{1}) (n_{i}-1)}-\frac{(n-1)(n-n_{i})}{n (n_{i}-1)} \\ &{} +\frac{(n-1)(n-n_{i}) (k-1)}{n (n_{i}-1)} \biggl[1-\frac{(2n-1)}{n-n_{1}}+\frac{n(n-1)}{(n-n_{1})^{2}} \biggr] . \end{aligned}$$

Let us consider a function

$$g(x)=1-\frac{(2n-1)}{n-x}+\frac{n(n-1)}{(n-x)^{2}},\quad 1\leq x\leq n-1. $$

Then

$$g^{\prime}(x)=\frac{1}{(n-x)^{3}} \bigl[(2n-1) x-n\bigr]>0. $$

Therefore $g(x)\geq g(1)=0$. Since

$$\frac{n-n_{1}}{n_{1}-1}\leq\frac{n-n_{i}}{n_{i}-1}\leq\frac{n-n_{k}}{n_{k}-1} $$

and $n_{k}\leq n_{i}\leq n_{1}$ $(1\leq i\leq k)$, from (15), we get

16 $$\begin{aligned} \rho_{1}(G)\leq{}&\frac{2(n_{1}-1)}{n-n_{1}}+\frac{n_{1} (n-1)^{2} (k-1)}{n (n-n_{1}) (n_{k}-1)}- \frac{(n-1)(n-n_{1})}{n (n_{1}-1)} \\ &{} +\frac{(n-1)(n-n_{k}) (k-1)}{n (n_{k}-1)} \biggl[1-\frac{(2n-1)}{n-n_{1}}+ \frac{n(n-1)}{(n-n_{1})^{2}} \biggr] , \end{aligned}$$

which gives the required result in (11). First part of the proof is done.

Now suppose that equality holds in (11). Then all the inequalities above must be equalities. From equality in (13), we get $x_{1}=x_{2}=\cdots=x_{k}$. From equality in (15), we get $n_{1}=n_{2}=\cdots=n_{i-1}=n_{i+1}=\cdots=n_{k}$. From equality in (16), we get $n_{i}=n_{1}=n_{k}$. From these results we conclude that $n_{1}=n_{2}=\cdots=n_{k}$.

Conversely, let $n_{1}=n_{2}=\cdots=n_{k}$. Then $n=n_{1} k$. Now,

$$\begin{aligned} &\frac{2(n_{1}-1)}{n-n_{1}}+\frac{n_{1} (n-1)^{2} (k-1)}{n (n-n_{1}) (n_{1}-1)}-\frac{(n-1)(n-n_{1})}{n (n_{1}-1)}+\frac{(n-1)(n-n_{1}) (k-1)}{n (n_{1}-1)} \\ &\qquad{} \times \biggl[1-\frac{(2n-1)}{n-n_{1}}+\frac{n(n-1)}{(n-n_{1})^{2}} \biggr] \\ &\quad=\frac{2(n_{1}-1)}{n-n_{1}}+\frac{(n-1)^{2}}{n (n_{1}-1)}-\frac {(n-1)(n-n_{1})}{n (n_{1}-1)}+\frac{(n-1) (k-1)^{2}}{k (n_{1}-1)} \times\frac {n_{1}(n_{1}-1)}{(n-n_{1})^{2}} \\ &\quad=\frac{2(n_{1}-1)}{n-n_{1}}+\frac{2(n-1)}{n}=\rho_{1}(G),\quad \mbox{by Lemma 4.2.} \end{aligned}$$

 □

## Lower bound on the second largest eigenvalue of the resistance-distance matrix of complete multipartite graphs

In this section we find a lower bound on the second largest eigenvalue of the resistance-distance matrix of complete multipartite graphs. For this we need the following result.

### Lemma 5.1

([15])

*Let* *A* *be a* $p\times p$ *symmetric matrix*, *and let* $A_{k}$ *be its leading* $k\times k$ *submatrix*; *that is*, $A_{k}$ *is the matrix obtained from* *A* *by deleting its last* $p-k$ *rows and columns*. *Then*, *for* $i=1,2,\ldots,k$,

17 $$ \lambda_{p-i+1}(A)\leq\lambda_{k-i+1}(A_{k})\leq \lambda_{k-i+1}(A), $$

*where* $\lambda_{i}(A)$ *is the* *ith largest eigenvalue of* *A*.

### Theorem 5.2

*Let* *G* *be a complete* *k*-*partite graph* $K_{n_{1}, n_{2},\ldots, n_{k}}$ *of order* *n* *with* $n_{1}\geq n_{2}\geq\cdots \geq n_{k}$. *Then*

18 $$\begin{aligned} \rho_{2}(G)\geq{}& \max_{1\leq i< j\leq k} \biggl[ \frac{(n+1) (n_{i}+n_{j})-2 (n+n_{i} n_{j})}{(n-n_{i}) (n-n_{j})}-\frac{(n-1) (n_{i}+n_{j})}{(n-n_{i}) (n-n_{j})} \\ &{} \times\sqrt{ \biggl(1+\frac{n_{i} n_{j}}{n^{2}} \biggr)-\frac{4 n_{i} n_{j}}{n (n_{i}+n_{j})}} \biggr] . \end{aligned}$$

### Proof

By Lemma 5.1, we have $\rho_{1}(G)\geq\max_{1\leq i< j\leq k} \rho^{\prime}_{1}$ and $\rho_{2}(G)\geq\max_{1\leq i< j\leq k} \rho ^{\prime}_{2}$, where $\rho^{\prime}_{1}$ and $\rho^{\prime}_{2}$ are given by

$$\left \vert \begin{matrix} (n_{i}-1) r_{i}-\rho & n_{j} r_{ij} \\ n_{i} r_{ij} & (n_{j}-1) r_{j}-\rho \end{matrix} \right \vert =0, $$

that is,

$$\rho^{2}-\bigl[(n_{i}-1) r_{i}+(n_{j}-1) r_{j}\bigr] \rho+(n_{i}-1) (n_{j}-1) r_{i} r_{j}-n_{i} n_{j} r^{2}_{ij}=0. $$

So,

$$ \rho^{\prime}_{1}=\frac{(n_{i}-1) r_{i}+(n_{j}-1) r_{j}+\sqrt{[(n_{i}-1) r_{i}-(n_{j}-1) r_{j}]^{2}+4 n_{i} n_{j} r^{2}_{ij}}}{2} $$

and

19 $$ \rho^{\prime}_{2}=\frac{(n_{i}-1) r_{i}+(n_{j}-1) r_{j}-\sqrt{[(n_{i}-1) r_{i}-(n_{j}-1) r_{j}]^{2}+4 n_{i} n_{j} r^{2}_{ij}}}{2}. $$

Now,

$$\begin{aligned} (n_{i}-1) r_{i}-(n_{j}-1) r_{j}&= \frac{2(n_{i}-1)}{n-n_{i}}-\frac {2(n_{j}-1)}{n-n_{j}} \\ &=\frac{2(n-1) (n_{i}-n_{j})}{(n-n_{i}) (n-n_{j})}. \end{aligned}$$

Using the above result, we get

20 $$\begin{aligned} &\bigl[(n_{i}-1) r_{i}-(n_{j}-1) r_{j}\bigr]^{2}+4 n_{i} n_{j} r^{2}_{ij} \\ &\quad=\frac{4(n-1)^{2} (n_{i}-n_{j})^{2}}{(n-n_{i})^{2} (n-n_{j})^{2}}+\frac{4 n_{i} n_{j} (n-1)^{2}}{n^{2}} \biggl(\frac{1}{n-n_{i}}+ \frac{1}{n-n_{j}} \biggr)^{2} \\ &\quad=\frac{4(n-1)^{2} (n_{i}+n_{j})^{2}}{(n-n_{i})^{2} (n-n_{j})^{2}} \biggl[ \biggl(1+\frac {n_{i} n_{j}}{n^{2}} \biggr)- \frac{4 n_{i} n_{j}}{n (n_{i}+n_{j})} \biggr]. \end{aligned}$$

Moreover,

21 $$\begin{aligned} (n_{i}-1) r_{i}+(n_{j}-1) r_{j}&= \frac{2(n_{i}-1)}{n-n_{i}}+\frac {2(n_{j}-1)}{n-n_{j}} \\ &=\frac{2[(n+1) (n_{i}+n_{j})-2(n+n_{i} n_{j})]}{(n-n_{i}) (n-n_{j})}. \end{aligned}$$

Using (20) and (21) in (19), we get the required result in (18). □

### Corollary 5.3

*Let* *G* *be a complete* *k*-*partite graph* $K_{n_{1}, n_{2},\ldots, n_{k}}$ *of order* *n* *with* $n_{1}\geq n_{2}\geq\cdots \geq n_{k}$. *Then*

$$ \rho_{2}(G)\geq \frac{(n+1) (n_{1}+n_{2})-2 (n+n_{1} n_{2})}{(n-n_{1}) (n-n_{2})}-\frac{(n-1) (n_{1}+n_{2})}{(n-n_{1}) (n-n_{2})} \sqrt{ \biggl(1+ \frac{n_{1} n_{2}}{n^{2}} \biggr)-\frac{4 n_{1} n_{2}}{n (n_{1}+n_{2})}}. $$

## Conclusion

In this paper, resistance distances in complete multipartite graphs are given via the standard electrical approach. Then eigenvalues of the resistance-distance matrix of complete multipartite graphs are studied, with emphasis being placed on bounds for the largest and second largest eigenvalues. However, up to now, the study on the eigenvalues of a resistance-distance matrix has still been in its infancy. Further study in this field is greatly anticipated.
